# The Use of Arsenous Acid Not Objectionable

**Published:** 1907-05

**Authors:** 


### The American Journal of Dental Science

The Official Organ of the Florida, Georgia and South Carolina State Dental Societies

**Third Series**

VOL. XXXVIII | MAY, 1907 | NO. 5

EDITORS

WM. GIRD BEECROFT, D. D. S., Madison. Wis. B. H. TEAGUE, D. D. S., Aiken, S. C.

Published on the 10th day of each month.
Subscription price $1.00 per year.
ENTERED AS SECOND-CLASS MATTER, MADISON, WIS.

Address all communications, both business and editorial, to
The Dental Publishing Company, Madison, Wisconsin

### THE USE OF ARSENOUS ACID NOT OBJECTIONABLE.

In recent years we have heard, on all sides, much condemnation of the use of arsenous acid, by those, who for years, have used it. Many of our best men have been most positive in their statements that to use this drug at this day and age is almost an act of criminal mal-practice. We regret indeed that many of these men have been so loud in their denunciation and condemnation of this preparation, that many of those who believe in its use still, and continue to use it, were almost afraid to acknowledge their adherence to the old lines of practice. This action upon the part of the members of the profession, and there were not a few, goes to show how readily we are carried away with something new, only to learn in time that we have overstepped the bounds of propriety and moderation in those things which are of decided merit. Why any in the profession should so suddenly turn from an old tried and true friend and become such ardent advocates of newer and less satisfactory methods, seems hard to understand. Why the writer, who has ever been so great a supporter of anesthesia of any kind in the relief of humanity, and who has been considered immoderate, by many, in his advocacy of the use of cocain, in this instance lifts up his voice to cry out against the thrusting aside of arsenous acid in the removal of pulps from teeth, may seem unexplainable to many, but we trust that our critics will realize that we can see value in things aside from anesthetic.

That cocain can and should be used in many, if not in most cases, is possibly true, but that there are many cases where arsenous acid should be used, to the exclusion of all else, is an indisputable fact. But that cocain is used in hundreds of cases where it is contra-indicated is also an indisputable fact. In taking up a discussion of question, it is done from the humanitarian standpoint. There are many cases where it is utterly impossible to use cocain without inflicting considerable pain in so doing, and there are many cases in which it is an utter impossibility to remove all the nerve tissue from the canals of the teeth by this method. It is the writer's belief that in all teeth, having small and tortuous canals, it is absolutely necessary to use arsenous acid in the destruction of the pulp tissue if the most satisfactory results are to be accomplished.

The satisfaction derived from its use after many years of the most potent services, backed by records of thousands upon thousands of teeth that have been handled in this way, and are doing the most valuable of service to their owners, is certainly evidence, which is unsurmountable as to the value of this drug. In fact, much of the success in root canal treatment of the past in dentistry has been due to the use of this most valuable of agents in the hands of the skilled as well as unskilled operator. The fact that thousands upon thousands of root canals which have been only partially filled have remained quiet for years and possibly throughout the entire lifetime of the patient without giving any signs of trouble, has been due to the preservative and mummifying properties of the arsenous acid used in the devitalization of these teeth. And it is the belief of the writer that many of those who have discarded this valuable drug will, in the near future, if they have not already, come to a realization of the mistakes they have made in discarding it for remedies which have been theoretically described as being more desirable, while the practical side remains yet to be proven. From the fact that arsenic is used, and is one of the most important or necessary adjuncts to embalming fluids in use today, and that it is used by the various taxidermists and those interested in the preservation of animal tissues, is one of the strongest reasons for again returning to its use.

The writer was very much gratified recently when at a recent meeting of one of our prominent dental societies this question was brought before the body and a vote was taken to ascertain how many of the men were using arsenous acid, and it was found that almost the entire membership was using it to a greater or less extent. This was all the more gratifying because within a year or two had a similar vote been taken few indeed would have been those who dared express themselves as advocates of that drug.

So let us not, too hastily, discard the old which has been found "tried and true," for the new, though theoretically the results be the most promising and the advocates be many.

In conclusiou let us reiterate by saying that both arsenous acid and cocain have their places in the destruction of the dental pulp. The cases should be selected for each. While cocain and pressure should be used where possible without causing pain and where all the pulp can be romoved, where pain would be inflicted or all fragments of the pulp can not be removed, arsenous acid must be used if the most gratifying results are to be obtained. When used arsenous acid should always be accompanied by cocain if the irritating activity of the arsenic is to proceed with the least amount of reflex disturbance. A word to those who have not used cocain combined with arsenic. Cocain retards the action of the arsenic, requiring a much longer period to produce the result, but permits of pulp destruction without the slightest disturbance if the application is properly made.

The usual time required is from a weed to ten days if applied directly to the exposed pulp.—The Dentists Magazine.

